# Taste sensitivity in anorexia nervosa: A systematic review

**DOI:** 10.1002/eat.22886

**Published:** 2018-07-08

**Authors:** Emma Kinnaird, Catherine Stewart, Kate Tchanturia

**Affiliations:** ^1^ Department of Psychological Medicine Institute of Psychiatry, Psychology and Neuroscience, King's College London London United Kingdom; ^2^ South London and Maudsley NHS Foundation Trust London United Kingdom; ^3^ Illia State University Tbilisi Georgia

**Keywords:** anorexia nervosa, eating disorders, taste, taste perception, taste threshold

## Abstract

**Objective:**

There is evidence for altered processing of taste in anorexia nervosa, particularly in the areas of reward processing and hedonic sensitivity. However, research on whether people with anorexia nervosa identify taste stimuli accurately, known as taste sensitivity, has yielded mixed findings. The objective of this study was to synthesize the literature on taste sensitivity in this disorder to provide a basis for future discussion on whether altered taste sensitivity may be also implicated in wider atypical taste processing in anorexia.

**Method:**

Electronic databases were searched systematically to identify published research examining taste sensitivity in anorexia. Search terms were “anorexia nervosa”, or “eating disorder”, combined with “taste”. 18 studies met inclusion criteria.

**Results:**

The review of the findings suggest that individuals with AN may experience reduced taste sensitivity that may improve following recovery. However, there was a significant variability in results across studies, potentially reflecting methodological problems including low sample sizes, experimental designs, and uncontrolled confounding variables. **Discussion**: This review suggests that altered taste sensitivity could represent a component in the wider altered taste processing observed in anorexia nervosa. However, the heterogeneity of findings highlight the need for future research to consider methodological issues raised by this review.

## Resumen

**Objetivo:**

Existe evidencia de una alteración en el procesamiento del gusto en la anorexia nervosa, particularmente en áreas del procesamiento de la recompensa y la sensibilidad hedónica. Sin embargo, la investigación sobre si las personas con anorexia nervosa identifican los estímulos del gusto con precisión, conocida como sensibilidad del gusto, ha arrojado resultados mixtos. El objetivo de este estudio fue sintetizar la literatura sobre la sensibilidad del gusto en este trastorno para proporcionar una base para la discusión futura sobre si la sensibilidad al gusto alterada puede estar implicada también en el procesamiento atípico, más amplio, en la anorexia.

**Método:**

Se realizó una búsqueda sistemática en bases de datos electrónicas para identificar investigaciones publicadas que examinaron la sensibilidad del gusto en la anorexia. Los términos de búsqueda fueron “anorexia nervosa” o “trastorno alimentario”, combinados con “gusto”. Un total de 18 estudios cumplieron los criterios de inclusión.

**Resultados:**

la revisión de los hallazgos sugiere que las personas con AN experimentaron una sensibilidad del gusto reducida que puede mejorar después de la recuperación. Sin embargo, hubo una variabilidad significativa en los resultados entre los estudios, lo que podría reflejar problemas metodológicos que incluyen tamaños de muestra pequeños, diseños experimentales y variables de confusión no controladas.

**Discusión:**

Esta revisión sugiere que la sensibilidad alterada del gusto podría representar un componente en el procesamiento del gusto alterado más amplio observado en la anorexia nervosa. Sin embargo, la heterogeneidad de los hallazgos resalta la necesidad de que las investigaciones futuras consideren las cuestiones metodológicas planteadas por esta revisión.

## 1 INTRODUCTION

Taste plays a key role in eating behavior (Boesveldt & de Graaf, 2017). Our ability to sense the primary tastes of sweet, salt, umami (savory), bitter, and sour enables us to identify the nutritional quality of food (van Dongen, van den Berg, Vink, Kok, & de Graaf, 2011). It has also been suggested that fat should be considered as a primary taste (Mattes, 2011). Taste additionally informs how pleasurable we find different foods: sweet tastes are generally perceived as more pleasurable, whereas bitter tastes are seen as unpleasant (Steiner, Glaser, Hawilo, & Berridge, 2001). This closely influences what foods we choose to eat, and in what quantities: in adults, the majority of our nutritional intake comes from predominantly sweet foods (47%), with only 14% of our calorie intake coming from foods rated as sour or bitter (Mattes, 1985). Consequently, taste is key in informing whether we find food rewarding, which in turn drives our appetite and eating behaviors through the brain reward system (Rolls, 2015).

Significantly, it has been suggested that altered reward processing may contribute towards the key symptoms of anorexia nervosa (AN) (Kaye, Fudge, & Paulus, 2009). AN is a condition characterized by a persistent restriction of food intake (APA, 2013). Vitally, recently proposed hypotheses suggest that atypical responses to taste stimuli could contribute towards this characteristic restriction (Kaye, Wierenga, Bailer, Simmons, & Bischoff‐Grethe, 2013). While taste is usually rewarding (Rolls, 2015), in individuals with AN restriction, rather than taste, becomes rewarding (Kaye et al., 2013), and tastes are perceived as less pleasant (Szalay et al., 2010). The exact mechanism behind this altered reward processing of taste is debated: one possibility is that individuals with AN have an altered sensitivity threshold when consuming pleasurable tastes (Kaye et al., 2013). It has also been proposed that the hedonic properties of taste stimuli (i.e., the extent to which someone likes a taste) remain intact but the motivation for the stimuli is reduced (Keating, Tilbrook, Rossell, Enticott, & Fitzgerald, 2012).

Therefore, a significant proportion of research documenting atypical taste processing in AN, particularly brain imaging‐based studies, has focused on taste–reward processing and the associated areas of taste hedonics and motivation (see Keating et al., 2012, for review). However, atypical taste processing is not limited to hedonic or reward responses (McCrickerd & Forde, 2016). A closely related area is that of taste sensitivity, referring to how accurately and how intensely we identify different taste stimuli. Taste sensitivity is a broad term and has been measured in the literature using a number of different approaches. Taste sensitivity incorporates research on taste recognition thresholds (the minimum concentration at which an individual can identify a taste), taste detection thresholds (the minimum concentration at which an individual can discriminate a taste from water, or a neutral substance), and subjective perceived taste intensity (individual perception of the intensity of a given stimulus).

Taste sensitivity is mediated by taste receptors on the tongue: different individuals have subtle differences in taste receptors, and the density of taste papillae, that influence taste sensitivity—which in turn influences what foods we perceive as pleasurable and choose to eat (Grimm & Steinle, 2011). For example, individuals who display a heightened sensitivity to bitter tastes may avoid more bitter tasting foods (Keller, Steinmann, Nurse, & Tepper, 2002). Taste sensitivity can also be influenced by dietary experience or environment, with individuals perceiving salty tastes as less pleasurable after following a low‐sodium diet (Bertino, Beauchamp, & Engelman, 1982). Consequently, atypical taste sensitivity has been implicated in a number of negative outcomes relating to eating behavior. This includes a loss of taste in the elderly contributing to malnutrition, (Schiffman & Graham, 2000), and an association between low taste sensitivity and obesity (Overberg, Hummel, Krude, & Wiegand, 2012). It has been hypothesized that atypical taste processing may contribute towards the restricted eating behaviors seen in anorexia nervosa (Frank, Shott, Keffler, & Cornier, 2016). Significantly, if taste sensitivity is dampened in AN, then this could contribute to the documented reduced reward appeal of taste stimuli in this population (Keating et al., 2012).

Consistent with this hypothesis are findings that individuals with AN exhibit a reduced number of taste papillae, which could potentially contribute to altered taste processing (Wockel, Hummel, Zepf, Jacob, & Poustka, 2007; Wockel, Jacob, Holtmann, & Poustka, 2008). Findings from neuropsychological studies also support the hypothesis that taste sensitivity perception could be altered in AN. Imaging studies suggest that individuals with AN suggest a different or reduced activation to taste stimuli in the insula compared to HC (Frank et al., 2016; Monteleone et al., 2017; Wagner et al., 2008). Similarly, a study using EEG techniques to investigate the effects of pleasant (sweet) and unpleasant (bitter) taste stimuli in individuals with AN compared to HC found that individuals with AN showed differing patterns of activation in response to the different stimuli compared to HC (Toth et al., 2004). Significantly, three of these studies used individuals either recovered from AN or of a normal weight, suggesting that altered taste processing could persist following weight restoration.

However, studies on whether taste sensitivity is implicated in AN have reached conflicting conclusions, potentially reflecting small sample sizes or different methods. Therefore, the aim of the current review is to explore and synthesize the current literature on taste sensitivity in AN.

## 2 METHOD

The systematic review was conducted in line with PRISMA guidelines (Moher, Liberati, Tetzlaff, Altman, & The Prisma Group, 2009).

### 2.1 Eligibility criteria

Studies examining taste sensitivity (defined as the ability to perceive taste stimuli) in AN were included in this review. Only journal articles published in peer reviewed journals reporting data were considered; case studies, conference abstracts, theoretical, and opinion papers were excluded. Only papers published in English were included.

### 2.2 Information sources and search

Electronic databases (PsychInfo, Scopus, PubMed, and Web of Science) were searched for papers up to and including January 2018. The search terms were anorexia nervosa, OR eating disorder, AND taste. Reference lists of published papers were also screened for eligible articles, yielding three additional papers.

### 2.3 Selection

**Figure 1:**
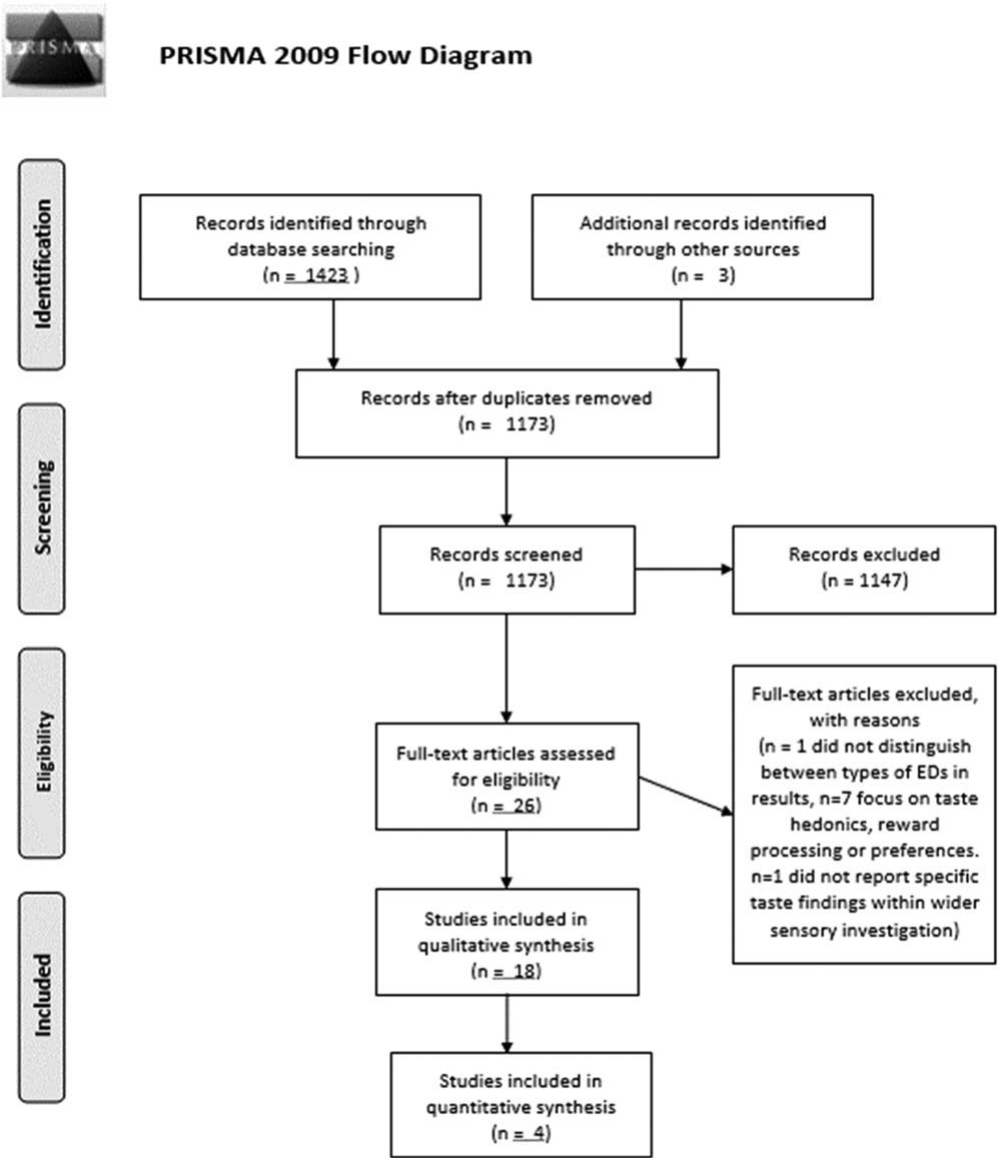
PRISMA diagram of study selection process

The selection process is summarized in Figure 1. Titles and abstracts of papers were screened for relevance to the topic of taste sensitivity. Papers exploring only reward–processing or hedonics of taste were not included. Full texts were obtained if the abstracts suggested that the paper was eligible, or if the eligibility was unclear. Any full texts which did not meet the inclusion criteria were excluded. Where papers reported multiple experiments or populations, only those sections relevant to AN and taste sensitivity are discussed.

Following study selection, data for the following variables was extracted independently from each paper: number of participants, age, BMI, gender, illness duration, diagnostic tool, how cases and controls were matched, exclusion criteria, design, methods, variables controlled for in analysis, and results. Where any information was unclear, the authors were contacted for clarification. The wide variation in methods identified in this review prevented the identification of any single summary measure for meta‐analysis, therefore only a qualitative synthesis is presented.

## 3 RESULTS

The systematic review identified a total of 18 studies measuring taste sensitivity, summarized in Table 1. Two papers (Fernandez‐Aranda et al., 2016; Ortega et al., 2016) were confirmed through correspondence with the authors to have reported on overlapping samples, with some of the cases and controls taking part in both studies. As this review did not incorporate a meta‐analysis, both studies were retained in the review, with the limitations and implications of their overlapping sample highlighted in the results and the discussion.

**Table 1:** Summary of studies included in systematic review

Methodology	Paper	Participants	Age (years) Mean (*SD*)	Mean BMI (*SD*)	Illness duration (years) Mean (*SD*)	Design	Equipment	Taste qualities Measured	Controlled variables (in exclusion, design or analysis)	Results
**Taste recognition**	Fernández‐Aranda et al. (2016)	AN (both R and B/P) = 64 HC = 80	24.0 (5.3) 22.6 (2.9)	17.4 (1.4) 21.6 (2.9)	5.5 (5.3)	Case–control	Taste recognition (taste strips)	Sweet, bitter, salty, sour	Age, gender, illness or medication that might affect taste, depression, smoking, contraceptive use, diabetes	**Sweet**: Lower sweet detection in AN (3.10 [0.15]) vs. younger HC (3.47 [0.13]) **Bitter**: No difference **Salty**: No difference **Sour**: No difference
	Ortega et al. (2016)	AN = 52 OB = 72 HC = 86	Overall sample 34 (12)	16.5 (1.3) 41.1 (7.7) 21.5 (2.8)		Case–control	Taste recognition (taste strips)	Sweet, bitter, salty, sour	Age, gender, illness or medication that might affect taste	**Sweet**: No difference **Bitter**: No difference **Salty**: No difference **Sour**: No difference
	Dazzi et al. (2013)	AN (both R and B/P) = 18 BN = 19 HC = 19	Overall sample 26.55 (6.29)	15.74 22.07 21.37		Case–control	Taste recognition (taste strips)	Sweet, bitter, salty, sour	Age, medical conditions affecting taste	**Sweet**: No difference **Bitter**: Lower bitter detection in AN compared to HC (6.33 [1.18] vs. 7.37 [0.91]) **Salty**: No difference **Sour**: No difference **Overall score**: Lower taste score in AN compared to HC (24.67 [3.67] vs. 27.79 [2.60])
	Goldzak‐Kunik et al. (2012)	AN = 15 HC = 15	15.8 (0.34) 15.0 (0.48)	17.2 (0.50) 19.4 (0.58)		Case–control	A selection of food tastes and nonfood tastes were prepared in concentrations of varying intensity and sprayed onto tongue. Scored on identification	Food tastes (apple, chocolate, cherry, chicken, and sweet vanilla) and nonfood tastes (toothpaste, coffee, chewing gum, soap, and infant vitamin drops)	Gender, school grade	**Overall score**: No significant differences across groups. Improved recognition of apple in AN, poorer recognition of chicken compared to HC
	Aschenbrenner et al. (2008)	AN‐R = 16 BN = 24 HC = 23	24.5 (4.0) 24.3 (4.6) 24.5 (4.8)	14.94 (2.05) 19.64 (3.05) 21.12 (2.20)		Case–control, longitudinal (admission/discharge)	Taste recognition (taste strips)	Overall taste scores given only	Gender, medication, medical conditions that might affect taste	**Overall score**: AN taste score on admission significantly lower compared to HC (22.25 [5.99] vs. 27.52 [3.06]). Significant improvement upon discharge (23.91 [3.65]). Taste scores correlated with BMI
	Nozoe et al. (1996)	AN = 9 HC = 6	19.3 (3.8) 21.8 (1.5)		21.4 (11.1) months	Case–control, longitudinal (admission, 1 week after initiation of treatment, when food intake reached 1600 kcal a day, discharge)	Filter paper disc method	Sweet, bitter, salty, sour	No systemic, endocrine, or central nervous system illness, medication, vomiting prior to admission, age, gender	**Sweet**: AN significantly lower compared to HC upon admission (exact scores not given). Did not significantly improve until discharge **Bitter**: AN significantly lower compared to HC upon admission (exact scores not given). Significantly improved when food intake increased **Salty**: AN significantly lower compared to HC upon admission (exact scores not given). Did not significantly improve until discharge **Sour**: AN significantly lower compared to HC upon admission (exact scores not given). Significantly improved when food intake increased **Overall score**: AN exhibited significantly poorer taste sensitivity upon admission compared to HC (97 [19] vs. 57 [7]). Significant improvement achieved when food intake increased (55 [15]) Significant improvement at discharge (46 [13])Taste scores not correlated with BMI or illness duration
	Jirik‐Babb and Katz (1988)	AN = 9 BN = 5 HC = 7				Case–control	Measuring recognition at different concentrations in distilled water	Sweet, bitter, salty, sour	Gender	**Sweet**: No difference **Bitter**: No difference **Salty**: No difference **Sour**: No difference
	Nakai et al. (1987)	AN = 23 BN = 13 HC = 18	19.3 (4.0) 21.5 (4.0) 22.0 (1.6)			Case control, longitudinal (7 AN retested following treatment).	Filter paper disc method	Overall taste scores given only (tested sweet, bitter, salty, sour)	Gender, no taste altering medication	**Overall score**: AN scores significantly lower compared to HC (9.9 [2.7] vs. 15.0 [1.8]). Improved following treatment but not to HC levels
	Casper et al. (1980)	AN = 30 HC = 10	19.1 (4.4) 22		2.3 (2.7)	Case control, longitudinal (7 AN retested following discharge).	Measuring recognition at different concentrations in distilled water	Overall taste scores given only (tested sweet, bitter, salty, sour)	Gender	**Overall score**: AN scores significantly lower compared to HC (11.8 [4.2] vs. 17.1 [1.4]). Improved significantly following discharge but not to HC levels No correlation with zinc plasma levels
	Casper et al. (1978)	AN = 13					Forced choice‐three stimulus drop technique.	Sweet, bitter, salty, sour	Gender	**Sweet**: Reduced recognition in AN **Bitter**: Reduced recognition in AN **Salty**: Reduced recognition in AN **Sour**: Reduced recognition in AN Participants with lower taste recognition had reduced plasma zinc levels, but some participants with normal taste recognition showed similarly lowered levels
**Taste Detection**	Eiber et al. (2002)	AN‐R = 20 AN‐BP = 20 BN = 20	23.3 (4.8) 26.4 (5.5) 27.5 (5.8)	15.7 (1.6) 16.1 (1.3) 22.7 (2.7)			Sweet taste perception threshold test: administered different sucrose solutions, reported when perceived sweet taste	Sweet	Gender, no taking psychotropic medication, noother DSM‐IV axis I diagnosis, no receiving parenteral nutrition, no medical condition interfering with taste, BMI	**Sweet**: AN‐R detection threshold significantly higher compared to AN‐BP/BN (no difference). Difference disappeared when BMI included as covariate
	Nakai et al. (1987)	AN = 23 BN = 13 HC = 18	19.3 (4.0) 21.5 (4.0) 22.0 (1.6)			Case control, longitudinal (7 AN retested following treatment).	Filter paper disc method	Sweet, bitter, salty, sour	Gender, no taste altering medication	**Sweet**: Reduced detection in AN **Bitter**: Reduced detection in AN **Salty**: Reduced detection in AN **Sour**: Reduced detection in AN
	Casper et al. (1980)	AN = 30 HC = 10	19.1 (4.4) 22		2.3 (2.7)	Case control, longitudinal (7 AN retested following discharge).	Measuring detection at from distilled water	Sweet, bitter, salty, sour	Gender	**Sweet**: Least affected in AN **Bitter**: Reduced detection in AN **Salty**: Less affected in AN **Sour**: Reduced detection in AN
	Lacey et al. (1977)	AN = 6 HC = 6	20.5 (5.1) 21.1 (6.9)			Case–control	Forced choice method: discriminating between distilled water and sucrose water at varying concentrations	Sweet	Age, gender, caloric intake	**Sweet**: No significant difference between AN (0.49 [0.50]) and HC (0.74 [0.50]), although AN trending lower than HC. Taste threshold of both anorectics and controls related to calorific intake
**Subjective Taste Intensity**	Frank et al. (2016)	AN‐R = 21 AN‐R Rec = 19 BN = 20 OB = 19 HC = 27	22.9 (6.1) 27.0 (5.3) 25.2 (5.3) 28.2 (8.1) 26.2 (7.0)	16.0 (1.1) 20.2 (1.1) 22.6 (5.7) 34.7 (4.6) 21.5 (1.4)		Case–control	Rating intensity of different concentrations	Sweet	Gender	**Sweet**: No difference
	Schebendach et al. (2015)	AN (both R and B/P) = 25 HC = 25	27.2 (7.8) 23.3 (3.5)	17.3 (2.0) 21.1 (1.6)		Case–control	Participants rated fat content of different cream cheese samples in their mouths	Fat	Gender, no smoking, age	**Fat**: No difference
	Goldzak‐Kunik et al. (2012)	AN = 15 HC = 15	15.8 (0.34) 15.0 (0.48)	17.2 (0.50) 19.4 (0.58)		Case–control	Tastes prepared in concentrations of varying intensity and sprayed onto tongueParticipants rated intensity	Sweet, bitter, salty, sour, umami	Gender, school grade	**Sweet**: No difference **Bitter**: No difference **Salty**: No difference **Sour**: No difference **Umami**: No difference
	Klein et al. (2010)	AN (both R and B/P) = 24 HC = 24	25.21 (1.08) 26.70 (1.4)	16.08 (0.25) 21.01 (0.66)	8.3 (1.4)	Case–control	Modified sham feeding technique to measure intake of five different solutions with differing levels of sweetness	Sweet	Gender	**Sweet**: No difference
	Simon et al. (1993)	AN‐R = 11 HC = 14	26.8	14 20.7	8.9	Case–control	Rating intensity of soft cheese stimuli modified for sweetness	Sweet	Gender, type of AN	**Sweet**: No difference
	Sunday and Halmi (1990)	AN‐R = 48 AN‐B/P = 36 BN = 42 HC = 26	AN‐R = 48 AN‐B/P = 36 BN = 42 HC = 26	18.94 (0.85) 21.31 (0.80) 21.51 (0.89) 19.27 (0.24)	14.68 (0.27) 16.30 (0.36) 20.75 (0.31) 20.91 (0.33)	Case–control, longitudinal (pre and post treatment)	Rated sweetness and fattiness intensity of different dairy solutions with varying levels of sweetness and fat. Also water taste test with varying levels of sweetness	Sweet, fat	Medication, type of AN	**Sweet**: No differences across groupsPerceived sweetness reduced following treatment **Fat**: AN‐BP rated fat more intensely compared to HC Perceived fattiness reduced following treatment in AN‐B/P only
	Jirik‐Babb and Katz (1988)	AN = 9 BN = 5 HC = 7				Case–control	Measuring magnitude estimation at different concentrations in distilled water	Sweet, bitter, salty, sour	Gender, duration of illness	**Sweet**: No difference **Bitter**: Lower intensity ratings for AN compared to HC **Salty**: Lower intensity ratings for AN compared to HC **Sour**: Lower intensity ratings for AN compared to HC Duration of illness correlated with lower intensity ratings
	Drewnowski et al. (1987)	AN‐R = 12 AN‐BP = 13 BN = 7 HC = 16	16.3 (2.2) 19.5 (4.2) 19.4 (2.5) 19.1 (0.8)	14.8 (1.6) 16.2 (2.3) 21.3 (2.0) 21.1 (1.6)		Case control (longitudinal, admission and post 3 week maintenance of target weight)	Rating sweetness and fat content of dairy stimuli with varying levels of sucrose and fat	Sweet, fat	Gender	**Sweet**: No difference **Fat**: No difference

The majority of studies used a case control design, with one study examining individuals with AN only (Casper, Kirschner, & Jacob, 1978), and one study comparing AN with bulimia nervosa (BN) but no healthy controls (HC) (Eiber, Berlin, de Brettes, Foulon, & Guelfi, 2002). Six studies additionally incorporated a longitudinal design, testing AN participants before treatment and/or following treatment or weight gain. Nine studies examined AN only, whilst nine additionally included either BN and/or obesity (OB). Only two studies included men in their analysis (Dazzi, De Nitto, Zambetti, Loriedo, & Ciofalo, 2013; Goldzak‐Kunik, Friedman, Spitz, Sandler, & Leshem, 2012), with one study not reporting participant gender (Sunday & Halmi, 1990), and all others using only women.

Overall, the review identified studies covering the tastes of sweet, salt, umami, bitter, sour, and fat, although not all studies covered all six tastes. A variety of different methods were used in these studies to measure taste sensitivity, with the absence of any one standardized measure prohibiting meta‐analysis. The studies used experimental approaches to measure the following aspects of taste sensitivity: taste recognition, taste detection, and subjective taste intensity. The results are summarized according to these four approaches.

### 3.1 Taste recognition

A total of ten studies identified in this review approached taste sensitivity by measuring taste recognition. All studies measured the ability of participants to recognize the presence of sweet, sour, bitter and salty taste stimuli, except for one study (Goldzak‐Kunik et al., 2012) which used commercially available preparations of different food and nonfood tastes (e.g., apple, chicken, toothpaste, coffee). Four studies (Aschenbrenner, Scholze, Joraschky, & Hummel, 2008; Dazzi et al., 2013; Fernandez‐Aranda et al., 2016; Ortega et al., 2016) used taste strips to measure taste recognition abilities for sweet, bitter, salty, and sour tastes (Mueller et al., 2003). This method involves the placing of strips of filter paper soaked in different taste solutions at four different concentrations on the tongue. The taste strips are presented at increasing concentrations but in a randomized order of tastes. Subjects are then asked to identify the taste after each strip, giving an overall taste score. A higher score indicates better tasting recognition. Two studies used the closely related filter paper disc method (Nakai, Kinoshita, Koh, Tsujii, & Tsukada, 1987; Nozoe et al., 1996), measuring the ability to recognize sweet, bitter, salty, and sour at different concentration thresholds (Berling, Knutsson, Rosenblad, & von Unge, 2011). Circular filter paper discs are soaked in different taste solutions at five different concentrations and placed on the tongue. Tastes are presented in an ascending order of concentrations, until the participant correctly identifies the taste. A points scoring system is used: 1 represents a correct answer at the lowest threshold, 5 a correct answer at the highest threshold. A score of 6 is given if the subject is unable to correctly identify the taste at any threshold. Therefore, a higher score indicates poorer tasting recognition. Other studies used unique experimental approaches to measuring taste recognition. These four studies used a similar approach in principle to the taste strips or filter paper disc methods, where individuals were presented with solutions of different taste qualities, sometimes of varying concentrations, and asked to identify the taste (Casper et al., 1978; Casper, Kirschner, Sandstead, Jacob, & Davis, 1980; Goldzak‐Kunik et al., 2012; Jirik‐Babb & Katz, 1988).

Findings on taste recognition in AN were mixed. Goldzak‐Kunik et al. (2012) found no differences across different food and nonfood tastes, but the fact that this was the only study to use food tastes (e.g., chocolate) as opposed to isolating specific taste qualities (e.g., sour, sweet), makes it difficult to compare and assess findings. The other nine studies assessed sweet, bitter, salty, and sour taste qualities, with inconsistent results. Six studies reported on the individual taste qualities: three out of these six studies found that individuals with AN had lower sweet taste recognition (Casper et al., 1978; Fernandez‐Aranda et al., 2016; Nozoe et al., 1996), three found that those with AN had lower bitter taste recognition (Casper et al., 1978; Dazzi et al., 2013; Nozoe et al., 1996), two found that those with AN had lower salty taste recognition (Casper et al., 1978; Nozoe et al., 1996), and two found that those with AN had lower sour taste recognition (Casper et al., 1978; Nozoe et al., 1996). Of the five studies reporting overall taste scores only, all studies found that individuals with AN had lower overall scores compared to HC (Aschenbrenner et al., 2008; Casper et al., 1980; Dazzi et al., 2013; Nakai et al., 1987; Nozoe et al., 1996).

Therefore, whilst the literature suggests that overall taste recognition may be lowered in individuals with AN compared to HC, studies are inconsistent on exactly which taste qualities are affected. To an extent this may reflect methodological issues. Sample power across the studies were typically small, with only one study using sample sizes of over *n* = 30 in every group (Fernandez‐Aranda et al., 2016; Ortega et al., 2016 discounted due to the sample overlapping with Fernandez‐Aranda). Additionally, unlike many of the studies in this review, Fernández‐Aranda et al. controlled for a number of potential variables in their study design, including smoking and medication use. Nonetheless, that a subsequent study using a similar design and an overlapping sample found no significant differences highlights the difficulty in replicating these findings (Ortega et al., 2016).

Moreover, not all studies controlled for confounding variables in their design or analysis. Only one study examining taste recognition distinguished between those with restrictive AN (AN‐R) and binge/purge AN (AN‐BP), with Aschenbrenner et al. (2008) including only those with AN‐R in their study. This is significant as taste could be potentially adversely affected by repeated vomiting (Rodin, Bartoshuk, Peterson, & Schank, 1990). Some other studies highlighted that they did not differentiate between people with AN‐R and AN‐BP in their analysis, but many did not specify if they included both subgroups, reporting the sample only as “AN”. Moreover, where studies did specify the inclusion of those with AN‐BP it was often unclear whether the diagnosis of purging was based on vomiting, or, for example, the use of laxatives or fasting. In line with this, only one study specified vomiting as an exclusion criteria (Nozoe et al., 1996). In addition, only a minority of studies controlled for smoking in their study design or analysis (Fernandez‐Aranda et al., 2016; Ortega et al., 2016). This is despite research suggesting that smokers exhibit lower taste sensitivity compared to nonsmokers (Chéruel, Jarlier, & Sancho‐Garnier, 2017).

Where studies did examine the role of potentially confounding variables, this was instructive in highlighting potential mechanisms behind these differences in taste recognition. Aschenbrenner et al. (2008) found that overall taste scores correlated with BMI. Although another study found that taste recognition did not correlate with BMI, this used a comparably small sample size (AN *n = *9) (Nozoe et al., 1996). In addition, studies that employed a longitudinal design, measuring individuals with AN both upon admission and discharge, found that taste recognition improved following treatment and discharge, although not normalizing to HC levels (Aschenbrenner et al., 2008; Casper et al., 1980; Nakai et al., 1987; Nozoe et al., 1996). Therefore, these findings suggest that lower taste sensitivity in AN could potentially improve, but not normalize, with treatment and/or weight restoration. However, the exact mechanisms behind these improvements remain unclear. Only one study controlled for illness duration in the analysis, finding that illness duration did not correlate with taste sensitivity (Nozoe et al., 1996). However, this may again reflect that study's small sample size.

### 3.2 Taste detection

Four studies measured taste detection thresholds, defined as the minimum concentration at which an individual can discriminate the presence of a taste stimulus from water, or a neutral substance (Casper et al., 1980; Eiber et al., 2002; Lacey, Stanley, Crutchfield, & Crisp, 1977; Nakai et al., 1987).

Whilst Casper et al.(1980) and Nakai et al.(1987) measured taste detection of sweet, bitter, salty, and sour taste qualities, finding reduced detection in people with AN, reporting standards were low, with both studies reporting reduced detection without giving exact scores.

Although Eiber et al. (2002) only measured sweet taste detection, the methods used in this paper was comparatively stronger. Despite not using a HC group as comparison, Eiber et al. (2002) was the only paper examining taste detection to distinguish between those with AN‐R and AN‐BP, finding that the detection threshold for those with AN‐R was significantly higher compared to those with AN‐BP and bulimia (BN). However, this difference disappeared when BMI was introduced as a covariate, suggesting that sweet taste detection thresholds may be related to a lower BMI. By comparison, Lacey et al. (1977) found no significant differences between the sweet detection thresholds of those with AN and HC. However, this was with a comparatively much smaller sample size, with *n = *6 in each group compared to *n = *20 in each group in Eiber et al. Nonetheless, Lacey et al. did find that taste thresholds of both those with AN and HC on a low calorie diet were comparatively lower compared to those on a high calorie diet, suggesting a relationship between taste sensitivity and diet.

### 3.3 Subjective taste intensity

Six studies measured perceptions of taste intensity by presenting participants with stimuli varying on a certain sweetness and/or fat and asking them to rate and compare the different stimuli on the intensity of this quality (Drewnowski, Halmi, Pierce, Gibbs, & Smith, 1987; Frank et al., 2016; Klein, Schebendach, Gershkovich, Smith, & Walsh, 2010; Schebendach et al., 2014; Simon, Bellisle, Monneuse, Samuellajeunesse, & Drewnowski, 1993; Sunday & Halmi, 1990). Two additional studies measured salty, bitter, and sour taste qualities in addition to sweet: Goldzak‐Kunik et al.(2012), and Jirik‐Babb and Katz (1988), presented these four different taste solutions in different concentrations, and asked participants to rate their intensity.

Different studies used different stimuli to explore subjective taste intensity. Three studies presented participants with tastes distilled at different concentrations in water (Frank et al., 2016; Goldzak‐Kunik et al., 2012; Jirik‐Babb & Katz, 1988). One study gave participants cherry Kool Aid solutions sweetened with varying concentrations of aspartame (Klein et al., 2010). The four other studies presented participants with dairy stimuli altered to have different concentrations of sweetness and/or fat (e.g., Schebendach et al., 2014; used fat free, low fat and regular types of the same cream cheese brand).

Specifically looking at sweetness and/or fat perception only, Drewnoski et al. (1988), Frank et al.(2016), Klein et al. (2010), Schebendach et al. (2014), Simon et al. (1993), and Sunday and Halmi (1990) and found that individuals with AN were equally as sensitive as HC in perceiving sweetness or fat. However, Sunday and Halmi (1990) did find that individuals with AN‐BP perceived solutions as fattier compared to controls before treatment, a difference which resolved following treatment. A key strength of this study was its sample size: whilst the other studies using this approach typically used small sample sizes, Sunday and Halmi (1990) included a total of 132 participants.

Of the two studies examining sweet, bitter, salty, and sour tastes, Goldzak‐Kunik et al. (2012) found no differences in perceived intensity across these taste qualities. Jirik‐Babb and Katz (1988) found no difference in perceived intensity of sweetness, though did find evidence for lower intensity ratings of bitter, salty, and sour tastes in those with AN compared to HC.

Consequently, the literature strongly suggests that perceived fat and sweetness intensity is not reduced or increased in AN when compared with HC, although there is some evidence that perceived fat intensity may be specifically increased in those with AN‐BP (Sunday & Halmi, 1990). This same study also suggests that this may resolve following treatment. These findings are supported by the fact that the majority of studies examining taste intensity did account for differences between AN subgroups, either dividing them into separate groups in the analysis, or specifying which subtype was included in the study design (typically AN‐R). For bitter, salty, and sour tastes, the literature is both limited and conflicting, with small sample sizes (total AN group is only *n = *24 across both studies) preventing further conclusions.

## 4 DISCUSSION

The overall findings of this systematic review indicate that the literature on taste sensitivity in AN is characterized by significant heterogeneity, potentially reflective of methodological limitations. Findings on subjective taste intensity were most consistent, with most studies suggesting that there are no differences in perceived sweetness and/or fat intensity between those with AN and HC. The majority of studies on taste recognition and taste detection suggested reduced thresholds in those with AN, although the significant disagreement across studies prevents any firm conclusions. Significantly, in studies that did find reduced taste recognition in AN and additionally incorporated a longitudinal design, results suggested that taste recognition may improve following treatment and discharge, but not normalize to HC levels.

This to an extent may indicate methodological limitations: sample sizes used across studies were often low, with the lowest study using *n = *6 per group (Lacey et al., 1977). Even where two studies used overlapping samples and the same experimental design, these found conflicting results, with one study reporting no differences (Ortega et al., 2016), and the other reporting lower sweet taste detection (Fernandez‐Aranda et al., 2016). Moreover, only a minority of studies accounted for variables which are known to affect taste, including smoking or repeated vomiting. In addition to methodological limitations, the variation in findings could also indicate that taste sensitivity in AN is characterized by a wide heterogeneity across individuals. If taste sensitivity does vary across individuals with AN then this suggests the need to control and identify potential variables in study design and analysis: for example, studies in this review suggest that taste sensitivity could be related to BMI, with a lower BMI relating to reduced taste sensitivity (Aschenbrenner et al., 2008; Eiber et al., 2002). Similarly, the findings that reduced taste recognition in people with AN may improve following weight restoration in recovery also require further research to isolate the potential causes of this improvement. The studies included in this review only explored this aspect from a longitudinal perspective, following the same patients from their illness state to weight restoration: future research could compare ill and remitted individuals to explore this issue further.

Only a minority of studies in this review explored potential biological mechanisms behind altered taste sensitivity in AN. Although Casper et al. (1978) found some evidence that reduced zinc plasma levels could be related to lower taste recognition in AN, a subsequent study with a larger sample size (Casper et al. 1980) did not support this finding. Moreover, despite evidence that the ability to taste some bitter compounds (e.g., 6‐n‐propylthiouracil, or “PROP”) is genetically determined, no study controlled for the potential of genetic variables, potentially contributing to the heterogeneity of results (Tepper, Banni, Melis, Crnjar, & Tomassini Barbarossa, 2014). Similarly, although a number of hormones, including leptin, cholecystokinin (CCK), and ghrelin, have been implicated in taste sensitivity, the reviewed studies did not assess hormone levels (Cai et al., 2013; Han, Keast, & Roura, 2017; Yoshida et al., 2017). Significantly, these hormones are known to be altered in AN, suggesting that future research could explore the role of these biological factors in taste sensitivity in this population (Atalayer, Gibson, Konopacka, & Geliebter, 2013; Cuntz et al., 2013; Hebebrand, Muller, Holtkamp, & Herpertz‐Dahlmann, 2006). Therefore, this systematic review highlights the need for controlled experimental designs in future research in taste sensitivity, with consideration for potential confounding variables, to illuminate the conflicting findings of previous research in this area.

The suggestion of some of the literature reviewed in this paper that individuals with AN may have reduced sensitivity resonate with previous research on taste and reward processing in this illness. Previous literature suggests that, unlike HC, individuals with AN may not process tastes as rewarding, contributing towards the characteristic symptom of food restriction (Kaye et al., 2013; Rolls, 2015). If taste sensitivity is indeed reduced in AN, then this could contribute towards this mechanism by reducing the pleasantness of food and so minimizing its reward value (Steiner et al., 2001). Consistent with this possibility is research suggesting that individuals with AN perceive tastes as less pleasurable (Szalay et al., 2010), although the research on this topic is again conflicting (Keating et al., 2012). If taste sensitivity is indeed reduced in AN, this could suggest that individuals with AN could benefit from interventions used to combat reduced taste sensitivity in other populations, such as introducing flavor‐enhanced foods into their diet to promote palatability and intake, or using zinc supplementation (Najafizade et al., 2013; Schiffman & Graham, 2000).

Interestingly, the hypothesis that individuals with AN have lowered taste sensitivity, or no differences in taste sensitivity compared to HC as suggested by the reviewed literature, conflicts with self‐reports of perceived heightened sensitivity in AN. Individuals with AN self‐report being hyper‐sensitive to taste stimuli, particularly sweetness or fat, and this may persist following weight restoration (Brand‐Gothelf et al., 2016; Pierce & Halmi, 1988). The findings of this study suggesting that there are in fact no differences in the perception of sweetness or fat intensity between people with AN and HC indicates that the documented aversion to this stimuli in individuals with AN reflects subjective perception, rather than objective taste alterations. Instead, sweetness and fat avoidance in AN may instead be driven by the cognitive resistance and inflexibility documented in this population (Lang, Stahl, Espie, Treasure, & Tchanturia, 2014; Tchanturia et al., 2012; Westwood, Stahl, Mandy, & Tchanturia, 2016).

Moreover, these findings indicate a potential conflict between self‐perceived (heightened) and actual (lowered) sensitivity to taste stimuli that closely resembles sensory prediction errors documented in AN in the field of interoceptive processing: individuals with AN appear to self‐report heightened levels of interoceptive sensitivity, whilst in fact exhibiting lower sensitivity on experimental measures (Khalsa et al., 2015; Khalsa & Lapidus, 2016). These interoceptive prediction errors have been associated with heightened anxiety and can act as a potent motivation to avoid the triggering stimulus‐ such as food or tastes (Kaye et al., 2004; Paulus & Stein, 2010). This could indicate that sensory prediction errors in AN are not isolated to interoception, but also exist in other, exteroceptive, sensory domains, such as taste, warranting further research.

## 5 LIMITATIONS

The key limitation of this systematic review was its inability to carry out a meta‐analysis on these findings, due to the lack of any consistent summary measure across the included studies. This reflected a wider difficulty in this review: it is likely that the wide variation in findings found in this review to an extent reflects the variation in methods used across studies, making direct comparisons difficult. However, this highlights the need for specific and highly controlled research designs in this field in the future, in order to both produce more reliable findings and to make comparison across studies easier.

## 6 CONCLUSIONS

This systematic review suggests that individuals with AN could experience lowered taste sensitivity. However, the previous literature on this topic is highly variable and characterized by methodological limitations. Future research in this area should consider the methodological issues raised by this review, including low sample sizes, experimental designs, and uncontrolled confounding variables, to explore whether these previous findings are replicable. Further research could also explore the potential mechanisms behind altered taste sensitivity in AN, including changes in BMI, diet, and biological factors.

## CONFLICT OF INTEREST

The authors have no conflicts of interest to declare.
